# A Novel In-Home Sleep Monitoring System Based on Fully Integrated Multichannel Front-End Chip and Its Multilevel Analyses

**DOI:** 10.1109/JTEHM.2023.3248621

**Published:** 2023-02-24

**Authors:** Shaofei Ying, Lin Wang, Yahui Zhao, Maolin Ma, Qin Ding, Jiaxin Xie, Dezhong Yao, Srinjoy Mitra, Mingyi Chen, Tiejun Liu

**Affiliations:** School of Life Science and TechnologyUniversity of Electronic Science and Technology of China 12599 Chengdu 610054 China; School of EngineeringThe University of Edinburgh 3124 EH8 9YL Edinburgh U.K; Department of Micor/Nano ElectronicsShanghai Jiao Tong University 12474 Shanghai 200240 China

**Keywords:** Biopotential acquisition front-end chip, in-home sleep monitoring, multi-scorer sleep staging, sleep architecture analysis

## Abstract

Objective: A novel in-home sleep monitoring system with an 8-channel biopotential acquisition front-end chip is presented and validated via multilevel data analyses and comparision with advanced polysomnography. Methods and procedures: The chip includes a cascaded low-noise programmable gain amplifier (PGA) and 24-bit $\Sigma $- $\Delta $ analog-to-digital converter (ADC). The PGA is based on three op-amp structure while the ADC adopts cascade of integrator feedforward and feedback (CIFF-B) architecture. An innovative chopper-modulated input-scaling-down technique enhances the dynamic range. The proposed system and commercial polysomnography were used for in-home sleep monitoring of 20 healthy participants. The consistency and significance of the two groups’ data were analyzed. Results: Fabricated in 180 nm BCD technology, the input-referred noise, input impedance, common-mode rejection ratio, and dynamic range of the acquisition front-end chip were $0.89 \mu $Vpp, 1.25 GN), 113.9 dB, and 119.8 dB. The kappa coefficients between the sleep stage labels of the three scorers were 0.80, 0.76, and 0.79. The consistency of the slowing index, multiscale entropy, and percentile features between the two devices reached 0.958, 0.885, and 0.834. The macro sleep architecture characteristics of the two devices were not significantly different (all p $>$ 0.05). Conclusion: The proposed chip was applied to develop an in-home sleep monitoring system with significantly reduced size, power, and cost. Multilevel analyses demonstrated that this system collects stable and accurate in-home sleep data. Clinical impact: The proposed system can be applied for long-term in-home sleep monitoring outside of laboratory environments and sleep disorders screening that with low cost.

## I. Introduction

Lack of good quality sleep can result in various economic, social, and health problems [1]. In order to understand patients suffering from sleep-related problems, long-term sleep monitoring has become an important clinical practice. The gold standard for sleep monitoring is attended in-lab polysomnography (PSG), which typically includes at least twelve biological signal channels. Moreover, three electroencephalogram (EEG) channels, two electrooculogram (EOG) channels, and one chin electromyogram (EMG) channel are basically included [2]. Sleep stages can be distinguished based on EEG, EOG and chin EMG signals, and the other channels are mainly used to detect events such as sleep apnea and leg movement [3], [4]. However, sleep disorders are episodic by nature and do not necessarily occur every night; thus, single-night PSG data may not be sufficiently informative. In addition, the high cost, lengthy waiting lists and discomfort experienced during laboratory sleep, limit the utility and diagnostic capability of PSG [5], [6], [7]. In particular, due to COVID-19, which can spread between people through close physical contact, many patients will not go to hospitals to receive care [8].

With the development of sensors and communication technologies, the field of sleep monitoring has gradually extended from in-lab to in-home environments. In-home sleep monitoring has gained considerable momentum in the field of sleep medicine due to its convenience and low costs, as well as its ability to mimic more realistic sleep conditions [9], [10], [11]. Moreover, sleep monitoring can be carried out by trained sleep technicians assisting participants with in-home portable devices [2]. To date, several in-home sleep monitoring methods, such as single-channel EEG [12], ballistocardiogram [13], photoplethysmography [14] and actigraphy [15], have been developed. Among them, single-channel EEG, such as UMindSleep [16], provides only coarse-grained information for sleep monitoring and thus cannot generate sufficiently accurate sleep assessment results. Another common sleep estimation method is actigraphy, which provides heart rate and movement information during sleep. Several publicly available off-the-shelf actigraphy-based products, such as Sleep Cycle [17], Apple Watch [18], and Fitbit [19], have been developed. These products have competitive advantages because of the deployment convenience and inexpensive. However, due to the lack of EEG information, these products cannot provide physiological details or sleep descriptions for diagnoses and thus cannot be applied to distinguish sleep stages. To the best of our knowledge, the portable and comfortable multichannel EEG in-home sleep monitoring system that can provide both fine-grained sleep assessments is still lack up to date. Moreover, no self-applied in-home sleep monitoring EEG system has been fully validated against PSG data, casting doubt on the clinical and research utility of this generation of devices.

Yu-Pin Hsu et al. [20] proposed an ultralow-power fully integrated front-end chip for ExG recording and performed basic EEG open-eye and closed-eye experimental tests. However, the multifaceted experimental demonstrations or scenario applications are not carried out. In addition, the ExG recording chip uses a 10-bit successive approximation register (SAR) analog-to-digital converter (ADC), resulting in a limited dynamic range (DR) and an input-referred noise (IRN); however, a greater sufficiently high DR and lower IRN may be required in complex in-home environments. To provide more natural sleep environments, reduce costs, and produce more accurate sleep assessments for patients [21], we propose a portable in-home sleep monitoring system based on a self-developed fully integrated multichannel biopotential acquisition front-end chip. The proposed chip is designed with an optimal trade-off based on the unique requirements of in-home system monitoring, including the size, weight, endurance, signal quality, noise resistance, etc. To develop such a portable device, we combined the single-chip system with multimodal signal monitoring. Meanwhile, we have a fully customized and optimized design for the sensing interface chip, thereby significantly reducing the system IRN and improving the input impedance, common-mode rejection ratio (CMRR), and DR. This guarantees high-quality physiological signals in a complex in-home environment for accurate distinguishes sleep stages.

## II. Circuit Design

**FIGURE 1.**
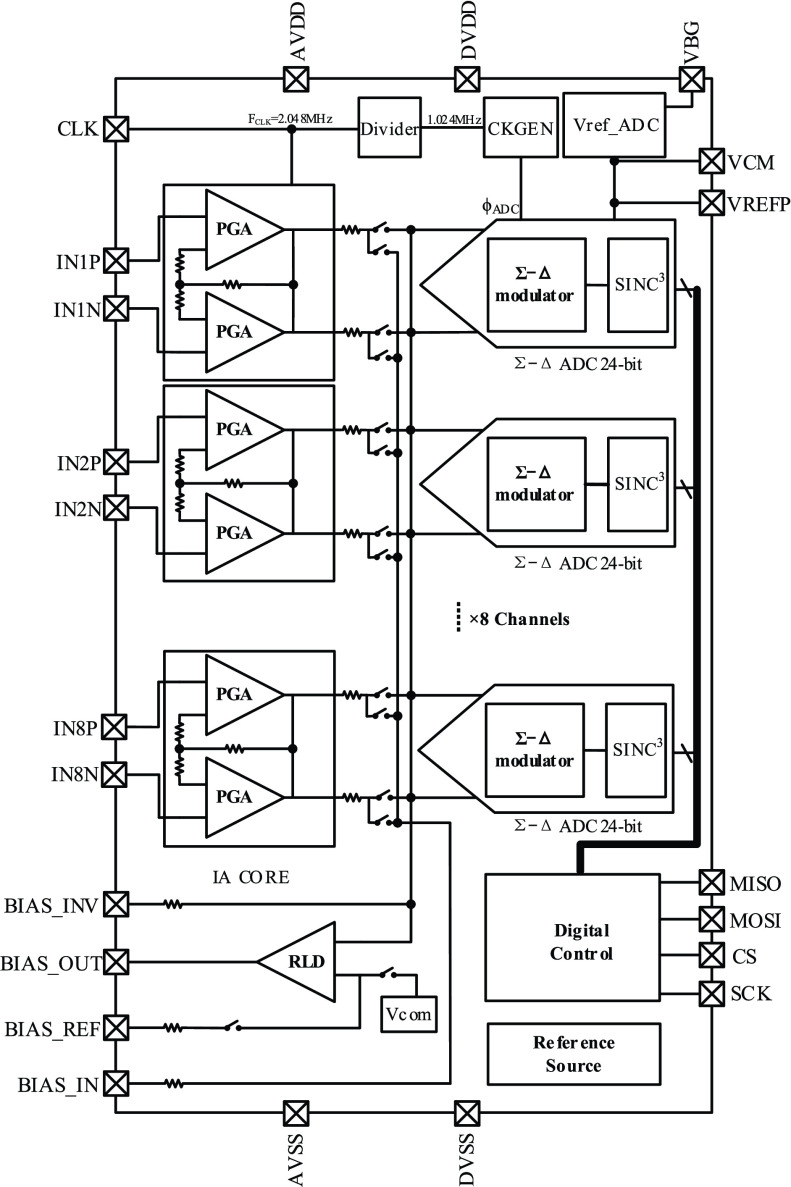
The architecture of the proposed fully integrated biopotential acquisition front-end chip.

An 8-channel biopotential acquisition front-end chip (ExG8CH-V1) with eight independent biopotential acquisition front-ends, a common-mode feedback amplifier, a low-noise reference source, and a digital controller was proposed. Fig. 1 shows the overall architecture of the chip. The eight independent fully integrated acquisition channels reduce the volume, power, and cost of the in-home sleep monitoring system.

Biopotential signals are collected independently by each channel, which includes a cascaded low-noise programmable gain amplifier (PGA) and 24-bit $\Sigma $- $\Delta $ ADC. The common-mode feedback amplifier applies a feedback bias voltage to the human body as a driven right leg (DRL). The low-noise reference source provides the required low-noise reference voltage and current. The digital controller writes the signal registers by each channel and reads the outputs of the ADC. To improve the signal quality of the in-home sleep monitoring system, low noise, high input impedance, large CMRR, and high DR are critical concerns when designing the biopotential acquisition front-end circuitry. The detailed implementation of the key blocks is described below.

### A. Low Noise, High Input Impedance PGA

The low-noise PGA is the first stage of the front-end circuit. This component, which is based on a three op-amp architecture, achieves high input impedance and a large CMRR [22]. The noise performance of the PGA determines the overall noise in the recording channel. Due to the frequency range of EEG signals (typically 0.1 Hz to 70 Hz), low-frequency flicker noise dominates the noise contribution. Therefore, chopper modulation was added to the input to modulate the low-frequency flicker noise to high-frequency noise, thereby reducing the noise in the frequency range of the EEG signal [23]. Compared to the auto-zeroing technique, chopper modulation minimizes the noise folding effect; thus, less noise is introduced. However, since chopper modulation introduces charging/discharging current into the input node, the input impedance of the front-end circuit tends to decrease. The reduced input impedance significantly attenuates the signal amplitude in non-invasive in-home sleep acquisition scenarios. Moreover, the reduced input impedances reduce the CMRR, which is proportional to the input impedance. The decreased CMRR causes the raw signal to be more affected by common-mode interference originating from interference at 50/60 Hz or other ambient noise interference.

To increase the input impedance and thus increase the CMRR, several impedance-boosting techniques were introduced. First, an auxiliary driving buffer was added to charge/discharge the input node before the chopper modulation technique was applied [24]. As a result, the input current introduced by chopper modulation was reduced, thereby increasing the input impedance. Second, positive feedback capacitance was added to cancel the parasitic capacitance of the input signal [25], [26]. This lower input capacitance increased the high-frequency input impedance. Finally, the custom-designed ESD diodes at the inputs were boot-strapped to ensure that their leakage current was minimized without significantly reducing the DC input impedance [27]. In addition to the aforementioned techniques, a DRL feedback loop was introduced to further increase the CMRR.

### B. High Dynamic Range 24-Bit $\Sigma$ – $\Delta$ ADC

During non-invasive in-home sleep monitoring, the recorded signal is susceptible to motion artifacts (MAs). This poses a significant challenge when designing the acquisition circuit. MAs are caused by relative movements between the skull and the dry electrodes. The amplitudes of most MAs are 5 to 6 orders of magnitude (up to volts) larger than those of the recorded biopotential signals (microvolts), thereby saturating the PGA. When the PGA is saturated by MAs, the biopotential acquisition channels cannot amplify weak biopotential signals. Back-end digital signal processing (DSP) can be utilized to remove MAs. However, the acquisition circuit should have a low gain and sufficiently wide DR. Therefore, an ultrahigh resolution, wide DR ADC should be developed for non-invasive multichannel biopotential acquisition.

**FIGURE 2.**
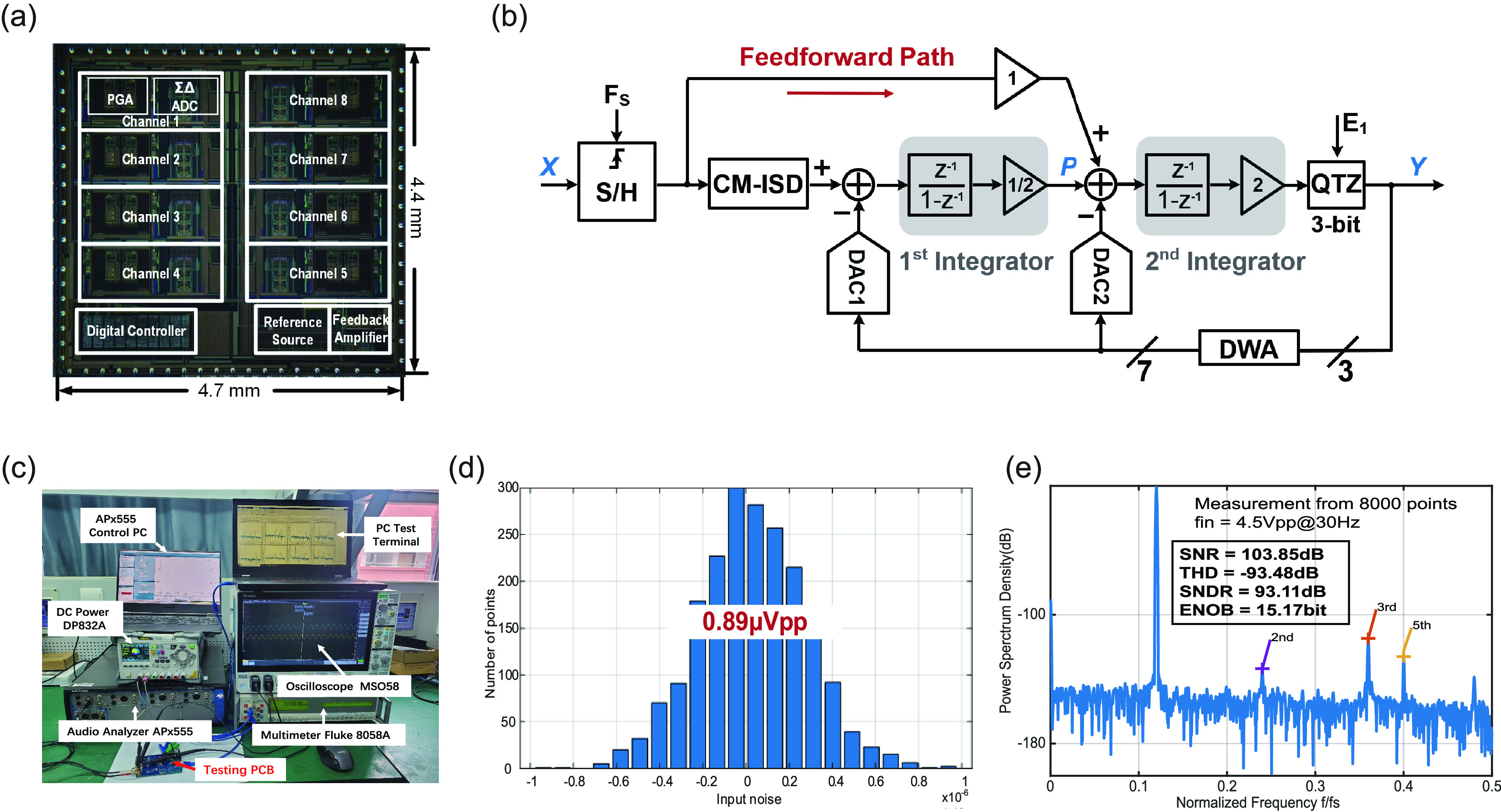
The proposed chip and system test. (a) Chip micrograph of the fabricated integrated front-end sleep monitoring system. (b) Detailed architecture of the proposed $\Sigma $- $\Delta $ modulator. (c) Testing PCB and automated measurement platform. (d) Input-referred noise histogram of the ICSMS. (e) Measured 8000-point FFT of the SNR, THD, SNDR, and ENOB of the ICSMS.

In the ExG8CH-V1 chip, the high DR 24-bit $\Sigma $- $\Delta $ ADC is based on a cascade of integrators feedforward and feedback (CIFF-B) architecture [28]. Fig. 2b shows the architecture of the proposed $\Sigma $- $\Delta $ modulator. The CIFF-B architecture reduces the output swing of the first integrator; thus, larger input amplitudes can be handled without significant distortion. An innovative chopper-modulated input scaling down (CM-ISD) technique is used to further attenuate the input amplitude. As a result, the maximum input of the modulator can be extended to the full scale without saturating or introducing instability in the loop. The mismatch in the feedback digital-to-analog converter (DAC) was reduced by the dynamic weighted averaging technique, thereby improving the signal-to-noise distortion ratio (SNDR). To effectively eliminate the excessive harmonic distortion and integration leakage caused by the parasitic capacitance of the input transistors, a cross-coupling positive feedback technique was employed in the feedforward Miller compensation transconductance amplifier (OTA). In addition, offset cancellation was used to reduce the input offset voltage of the multi-bit comparator, and the bubble removal technique was applied to prevent random errors in the output digital codes.

### C. Implementation Results

The ExG8CH-V1 chip has been fabricated with 5 V, 180 nm BCD technology, occupying a core area of 20.7 mm^2^ (4.7 mm $\times4.4$ mm). Fig. 2a shows the ExG8CH-V1 chip micrograph. The SNDR and SFDR measured by the $\Sigma $- $\Delta $ modulator are 110 dB and 115 dB, respectively, for a 30 Hz full-scale input. In addition, the proposed CM-ISD technique improves the DR from 99 dB to 124 dB. Table 1 compares the performance of the ExG8CH-V1 chip to the performance of chips in state-of-the-art works. Based on the custom-designed multichannel front-end chip, we developed a portable integrated circuit sleep monitoring system (ICSMS) that can be used for simultaneous EEG/EOG/EMG signal collection at in-home environments. Fig. 2c shows the PCB of the system and the automated measurement platform, and Fig. 2d, e depict the IRN, signal-to-noise ratio (SNR), total harmonic distortion (THD), SNDR, and ENOB (under a 500 Hz sampling rate) of the ICSMS, which have the values of $0.89 \mu $Vpp, 103.85 dB, −93.48 dB, 93.11 dB, and 15.17 bits, respectively.

**TABLE 1:** Comparison table with the state-of-the-art designs.

	JSSC’15 [29]	SSCL’18 [30]	TBCAS’18 [31]	TBCAS’21 [32]	JSSC’20 [20]	ADS1299’12 [22]	This work
Topology	IA+ $\Delta \Sigma $	VCO-based	IA+ $\Delta \Sigma $	$\Delta \Sigma $− $\Sigma $	PGA+SAR	IA+PGA+ $\Delta \Sigma $	IA+PGA+ $\Delta \Sigma $ IA+PGA+ $\Delta \Sigma $ IA+PGA+ $\Delta \Sigma $
Technology(nm)	180	65	180	180	180	N.A.	180
IRN( $\mu $Vpp)	4.0	14.5	7.9	6.5	8.05	0.98	0.89
SNR(dB)	83	N.A.	N.A.	105.6	57.6	121	103.9
DR(dB)	84.3	N.A.	N.A.	108.3	75	119.5	119.8
CMRR(dB)	>100	77	100	109	100	120	113.9
Resolution(bit)	16	10	15	24	10	24	24
Input impedance( $\Omega $)	>500M	>500M	720M	34M	N.A.	1G	1.25G
Area(mmB2/ch)	3.5	0.01	1.7	0.48	0.72	N.A.	2.6
Power(mW)	0.056	0.003	0.043	0.074	0.012	39	34.32
Application	ECG	ECoG	EEG	EEG/ECG	APW/EEG/EOG/EMG	EEG/ECG	EEG/EOG/EMG

The system integrates five-channel EEG, two-channel EOG, and one-channel EMG measurements. The system has a total weight of 30.3 g (18.9 g without the battery), a size of $64 \times 45\,\,\times31$ mm (length $\times $ width $\times $ height), and a power consumption of 89 mW (with a working time of more than 24 continuous hours); thus, the system can be placed on a participant’s pillow or inserted into their pocket without interfering with their sleep activity. In addition, the discrete data collected by the system can be stored online and offline simultaneously by Bluetooth wireless transmission and trans-flash card local storage, thereby guaranteeing that the experimental data are stored. Furthermore, we developed sleep monitoring software for the system based on C++ code and Qt software. The software allowed for real-time viewing of the sleep data waveforms, filtering and other processing. In conclusion, the overall performance of the ICSMS meets the requirements of non-invasive multichannel biopotential acquisition for in-home sleep monitoring.

## III. Materials and Methods

### A. Participants

Twenty young adults (15 males, 5 females) aged 24.95 ± 1.53 years were recruited from the University of Electronic Science and Technology of China (UESTC) to participate in this in-home sleep monitoring study. The participants reported normal sleep of approximately 7–9 hours per night, typically between 23:30 and 08:30. Moreover, the participants reported that they were healthy, took no medications, and had no history of sleep disorders or psychiatric/neurological conditions. In addition, participants were asked to abstain from alcohol, medication, caffeine, naps, and vigorous exercise starting 24 hours before their participation until the end of the two-night sleep study. To eliminate the first-night effect, sleep data recordings were collected in standard laboratory conditions after habituation during a night of undisturbed baseline sleep. This experiment was conducted according to the guidelines of the Declaration of Helsinki and was approved by the Ethics Committee of UESTC.

### B. Polysomnography and Manual Scoring

Two consecutive nights of in-home sleep monitoring experiments were performed using two devices: the ICSMS system proposed in this paper and the commercial actiCHamp amplifier (Brain Products, Germany). The public technical parameters of actiCHamp are as follows: input-referred noise $\leq 2 \mu $Vpp, CMRR $\geq100$ dB, input impedance $\geq $ 1 $\text{G}\Omega $, and weight of 1100 g. The ICSMD and actiCHamp recordings included five EEG channels (F4-M1, C4-M1, P4-M1, T4-M1, and O2-M1, according to the international 10–20 system, with the M1 and M2 electrodes placed on the left and right ear lobes), two EOG channels (E1-M2 and E2-M2, placed above the right and below the left outer canthus) and a chin EMG channel, with a sampling rate of 500 Hz. The PSG data were converted to EDF format, and sleep staging (blind analyses for both groups) was performed on Philips Sleepwear G3 software by three professional sleep physicians. Then, three scorers classified the 30 s epochs into non-rapid eye movement (NREM, stages N1, N2, or N3), rapid eye movement (REM), or wakefulness (W) using the most recent American Academy of Sleep Medicine (AASM) scoring criteria. During the staging process, the scorers were not informed which device collected the data. In addition, the scorers verbally confirmed that the two devices did not interfere with the sleep staging process. PSG recordings from two participants were not scored due to persistent high-frequency artifacts; therefore, recordings from 18 participants were used in the analyses.

### C. Data Processing and Analysis

The PSG data were processed offline using EEGLAB (Delorme & Makeig, 2004) and Python (Rubinov & Sporns, 2010). An infinite impulse response bandpass filter (0.3-35 Hz for EEG/EOG and 10–100 Hz for EMG) was applied to the time domain signals; then, all signals were segmented into 30 s epochs. Next, feature extraction and computation of all PSG data were performed using the Luna C/C++ pipeline developed by S.M.P. (https://zzz.bwh.harvard.edu/luna/). The consistency (intraclass correlation coefficient) and significance (p-value) were determined with statistical software, including SPSS 26 (SPSS Inc., Chicago, IL) and GraphPad Prism 8 (GraphPad Software Inc., CA). When the data were not disturbed, the three scorers did not consider the P4-M1 and T4-M1 channels, and the relative amplitude of the EMG signal was considered only as an auxiliary reference for staging; thus, the F4-M1, C4-M1, O2-M1, E1-M2, and E2-M2 channels were used in subsequent demonstrations.

#### 1) Power Spectrum Density

The time domain data were subjected to power spectrum density (PSD) analyses using a modified version of the Welch periodogram method (Tröbs & Heinzel, 2006) with overlapping windows (4 s windows with 2 s overlap). The PSDs ( $\mu \text{V}^{2}$/Hz) were estimated over a continuous range from 0.5 to 30 Hz in steps of 0.5 Hz. Frequencies less than 0.5 Hz and greater than 30 Hz were not included to prevent slow artifacts arising from skin potentials, and the power spectral density beyond 30 Hz was negligible. Then, the frequency spectra were transformed to log-log coordinates to calculate the power-law frequency scaling exponent (slope of the line of best fit for frequencies between 0.5 and 30 Hz), which was estimated using simple linear regression. Moreover, the relative power spectrum density (RELPSD) was determined in the following frequency bands: slow (0.5-1 Hz), delta (1-4 Hz), theta (4-8 Hz), alpha (8-12 Hz), sigma (12-15 Hz), and beta (15-30 Hz). The RELPSD was calculated by dividing the absolute band power by the total power. In addition, we calculated an EEG/EOG slowing index, which was defined as si = f(f(f(relative delta power) + f(relative theta power))/f(f(relative alpha power) + f(relative beta power))), where f(x) is a function that indicates that outliers (+/−3 standard deviations) are set to missing.

#### 2) Multiscale Entropy

Entropy is a nonlinear dynamic parameter that reflects the complexity of a system. Since the brain is a nonlinear dynamic system, some researchers have found that multiscale entropy algorithms utilizing multiple timescales can detail changes inherent in brain EEG signals [33]. Multiscale entropy (MSE) extends the idea of sample entropy (SE) to multiple time scales and is an effective method for quantifying the complexity of a time series over different time scales. This research is based on the SE/MSE estimation method described by Costa et al. [34]. Briefly, this method has two steps: first, the time series is coarse-grained, which depends on scale parameters (taking 1, 3, 5, 7, and 9 in this calculation); then, the SE is calculated for each coarse-grained time series, which depends on the parameters m (the pattern length, set to 2.0) and r (the similarity criterion, set to 0.15).

#### 3) Percentile Statistical Distribution

In statistics, the percentile, which is commonly used in descriptive analyses of data bureaus, is a positional indicator used to present information about data distributions between the minimum and maximum values. The percentile is defined as a set of data containing n values sorted from smallest to largest, with the value at the P% position called the Pth percentile. We standardize the signal using a robust approach, namely, the measures of central tendency; then, we spread the median and estimate the standard deviation (SD) based on the interquartile range. Finally, the following percentiles were calculated: P01, P02, P05, P10, P20, P40, P60, P80, P90, P95, P98, and P99.

#### 4) So/Spindle Detection

Sleep spindle activity and slow oscillations (SOs) are notable features of sleep EEG during the NREM sleep stage. We use a wavelet-based sleep spindle detection algorithm in Docker Luna C/C++ and perform automatic detection at two target frequencies corresponding to the center frequencies of the Morlet wavelet for slow spindles (Fc = 11 Hz) and fast spindles (Fc = 15 Hz). After bandpass filtering the signal for Fc ±2 Hz, the magnitude of the wavelet transform was smoothed (sliding window of 0.1, win) and scaled according to the stage signal mean. A spindle is identified when a core interval of at least 0.3 seconds exhibits a signal with an amplitude of at least 4.5 times the mean. Furthermore, a spindle must exhibit an extended interval of at least 0.5 seconds with a signal at least 2 times the mean. For SO detection, we first applied bandpass filtering from 0.5 to 4.0 Hz using a linear phase finite impulse response filter. Then, we followed the criteria published by [35], including (1) a negative peak amplitude of at least $- 40\,\,\mu \text{V}$, (2) a peak-to-peak amplitude of at least $75 \mu \text{V}$, (3) a negative wave deflection duration between 0.3 s and 1.5 s, and (4) a positive wave deflection duration between 0 s and 1 s.

#### 5) Statistical Analysis

A desirable measure of reliability should reflect both degrees of correlation and agreement between measurements, that intraclass correlation coefficient (ICC) is such as an index [36]. In this research, for the single scorer sleep staging results, the consistency between the micro sleep architecture features of the sleep data collected by the two devices was assessed using the ICC. The ICC estimates and their 95% confident intervals were calculated by the SPSS statistical package based on a single measurement, consistency coefficient, and a two-way mixed effects model. According to the guideline for reporting the ICC, the values less than 0.5, between 0.5 and 0.75, between 0.75 and 0.9, and greater than 0.90 are indicative of poor, moderate, good, and excellent reliability, respectively [36], and that most of them are greater than 0.75 in this paper. Furthermore, because the macro sleep architecture features were not normally distributed, these data were analyzed by non-parametric Mann-Whitney U tests.

## IV. Results

### A. Multi-Scorer Sleep Staging Results

**FIGURE 3.**
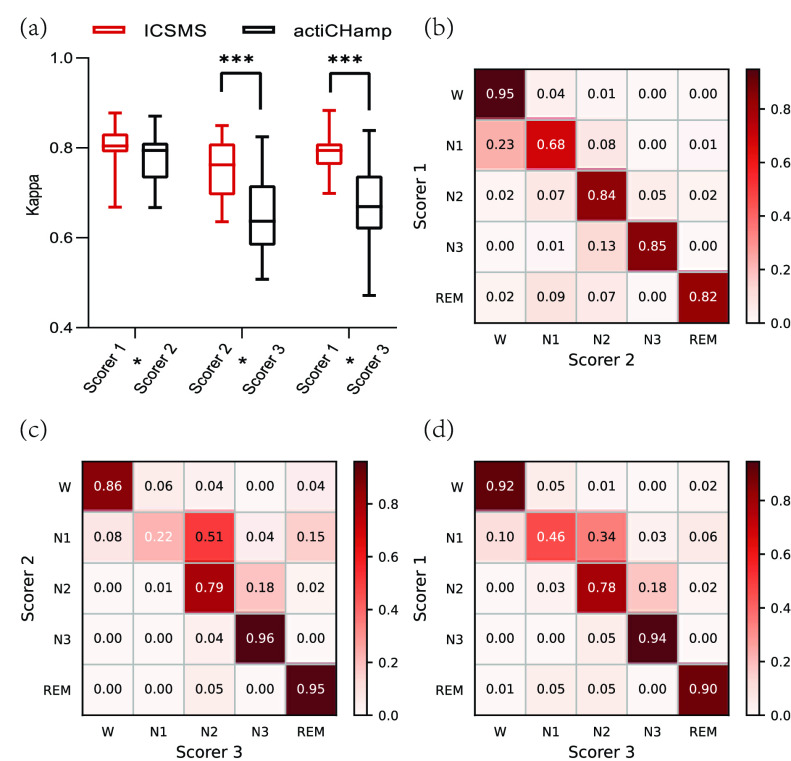
Consistency of the sleep staging results of the three scorers. (a) Kappa coefficients for sleep staging results (including W, N1, N2, N3 and REM staging) of the three scorers, with a total of 20,152 labels for the 18 participants. The orange and black solid curves indicate the ICSMS and actiCHamp devices, respectively. (b), (c), (d) Confusion matrices of the ICSMS staging labels for the three scorers’ cross validation. ***, p < 0.001.

Fig. 3 depicts the correlation among the staging results of the three scorers. Fig. 3a shows the Cohen’s kappa coefficients of the sleep staging results between the three scorers (from the ICSMS and actiCHamp devices). The kappa coefficients are almost greater than 0.6, which suggests considerable consistency in the sleep staging results of each device among the three scorers. Moreover, we observed no significant difference by independent samples t-test in kappa coefficients between scorers 1 and 2 (t = 1.333, p = 0.191; mean ± SD are 0.80 ± 0.05 for ICSMS and 0.78 ± 0.06 for actiCHamp). However, there were significant differences between scorers 2 and 3 (t = 4.158, p < 0.001; mean ± SD are 0.76 ± 0.06 for ICSMS and 0.65 ± 0.09 for actiCHamp) and scorers 1 and 3 (t = 4.593, p < 0.001; mean ± SD are 0.79 ± 0.04 for ICSMS and 0.68 ± 0.09 for actiCHamp). The kappa coefficient of the ICSMS device was significantly higher than that of the actiCHamp device between scorers 2 and 3 and scorers 1 and 3, which may be due to the better stability of the sleep data collected by the ICSMS device, thereby reducing the impact of the scorers’ staging.

Furthermore, Figs. 3b, c, and d show confusion matrices for the consistency of the three scorers’ staging results according to the data collected by the ICSMS device. The W, N2, N3, and REM stages were accurately identified; however, the most difficult state to identify was N1, which may be due to the fact that this stage had few examples and is the least distinctive. The Cohen’s kappa coefficients and confusion matrix results demonstrate that the ICSMS device collects highly stable data and that these data do not pose difficulties for scorers’ staging.

### B. Micro Sleep Architecture

In this section, the EEG/EOG features are shown, and intraclass correlation coefficients are used to indicate consistency among the data features collected by the ICSMS and actiCHamp devices. For all statistical analyses, the data are presented as the mean ± SD. The results of scorer 1 are shown, and the results of the other two scorers are included in the supplementary materials (Fig. S2. to Fig. S13.).

#### 1) PSD Slopes in Different Sleep Stages

**FIGURE 4.**
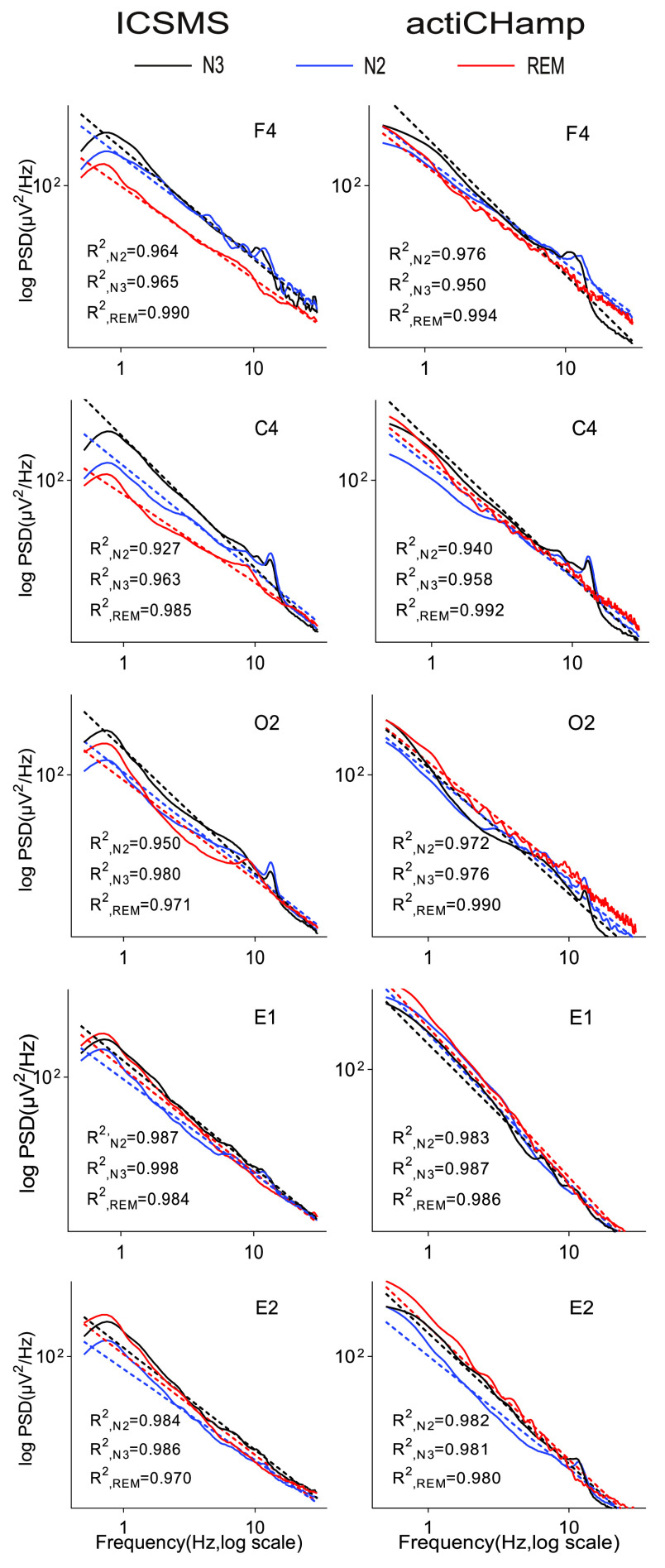
Average power spectral density (PSD) plots (on a log-log scale), shown separately for each sleep stage and channel, as well as robust fits (dashed lines, calculated by using simple linear regression) and the determination coefficient $\text{R}^{2}$.

It is well established that, in specific ranges, the background frequency spectra of electrophysiological mass activities, including scalp EEG, follow power-law distributions, with the power inversely proportional to the frequency (1/ ${f}$) according to a scaling exponent [37]. The coefficients of determination $\text{R}^{2}$ of all PSD slopes were calculated, and the average values across all channels and sleep stages were 0.974 and 0.976 for the ICSMS and actiCHamp devices, respectively, indicating that the data collected by the ICSMS device have linear PSD slopes. As shown in Fig.4, the PSDs were increasingly more negatively sloped from N2 to N3 stage and decreased in the REM stage (except for E1 and E2, which is the most significant feature of the REM stage). The results of the repeated measures ANOVA test for the PSD slopes of the three stages showed significant differences across sleep states (all pairs ps < 0.05). In addition, changes in the low frequency bands (slow and delta bands) across sleep stages were considerably more pronounced than the increases in the higher frequency bands, with the PSDs gradually decreasing from N3 to N2 to REM, while the PSDs increased in the spindle band during the N2 and N3 stages. These results are consistent with trends in sleep stage changes [38]. The statistical results show log-linear relationships between the PSD and frequency in each stage, which is consistent with the power-law distribution.

#### 2) RELPSD and EEG/EOG Slowing Index In Different Sleep Stages

**FIGURE 5.**
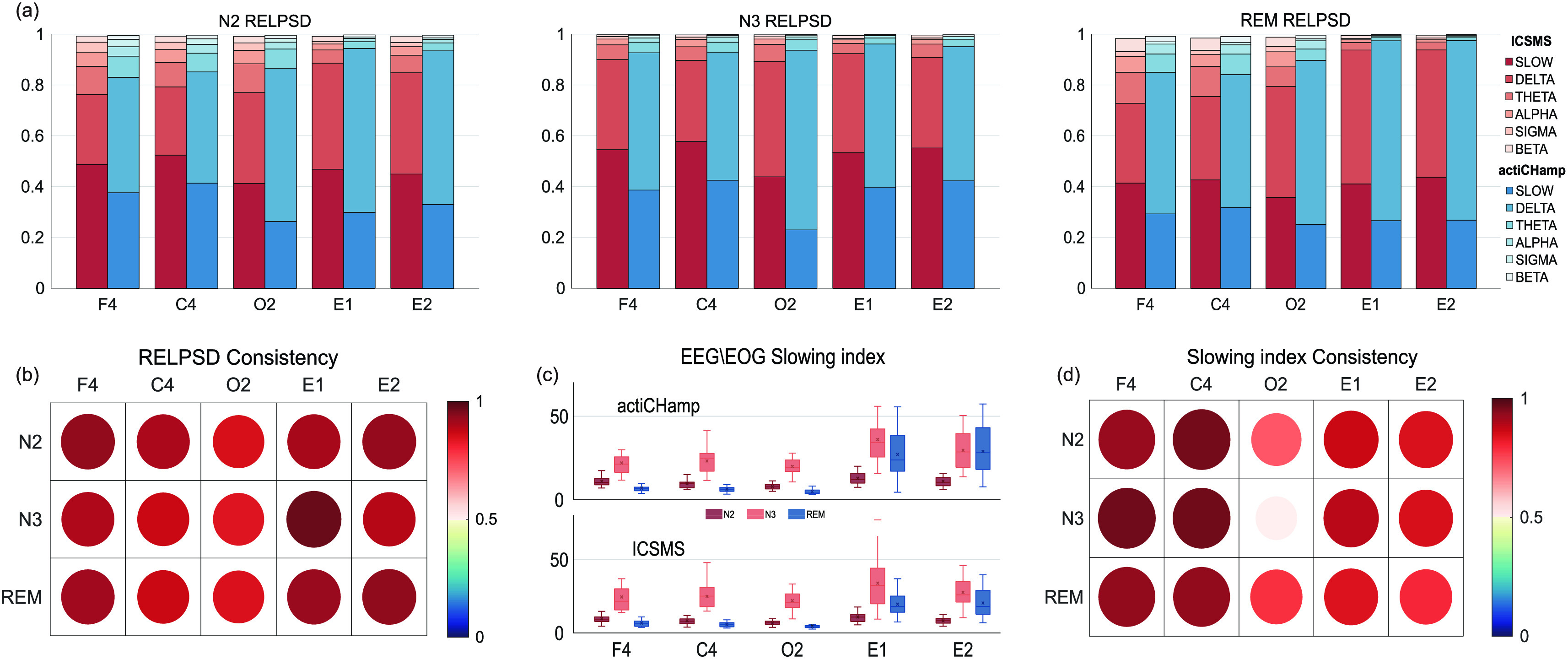
The relative power spectrum density (RELPSD) and EEG/EOG slowing index. (a) N2, N3, and REM stage RELPSD value stack for the slow, delta, theta, alpha, sigma, and beta bands for each device. (c) EEG/EOG slowing index distribution for each stage and channel of the two devices. (b) and (d) RELPSD and EEG/EOG slowing index consistency between the ICSMS and actiCHamp devices.

Fig. 5a shows the EEG/EOG RELPSD data collected by each device in each channel and frequency band (slow, delta, theta, alpha, sigma, and beta), averaged across the three sleep stages. The average RELPSD of slow, delta and theta for all channels show occupies the percentage of an absolute part. Fig. 5c shows the distribution of the EEG/EOG slowing index for each stage, and the data collected by the two devices show remarkable consistency. The ICC values of the RELPSD and EEG/EOG slowing index between the two devices are shown in Fig. 5b, d, where the consistency values of each stage and channel were 0.913 ± 0.033 (with 95% CI = 0.773-0.989; all p < 0.0001) and 0.852 ± 0.12 (with 95% CI = 0.428-0.975; all p < 0.005), respectively, and no values were less than 0.75. Moreover, after combining all stages and channels data into a sequence, the ICC values of the RELPSD and EEG/EOG slowing index reached 0.973 (with 95% CI = 0.970-0.975; all p < 0.0001) and 0.958 (with 95% CI = 0.951-0.979; all p < 0.0001), respectively, indicating that the data collected by the ICSMS system are highly consistent with the data collected by the actiCHamp system.

#### 3) Percentile Distribution in Each Channel

**FIGURE 6.**
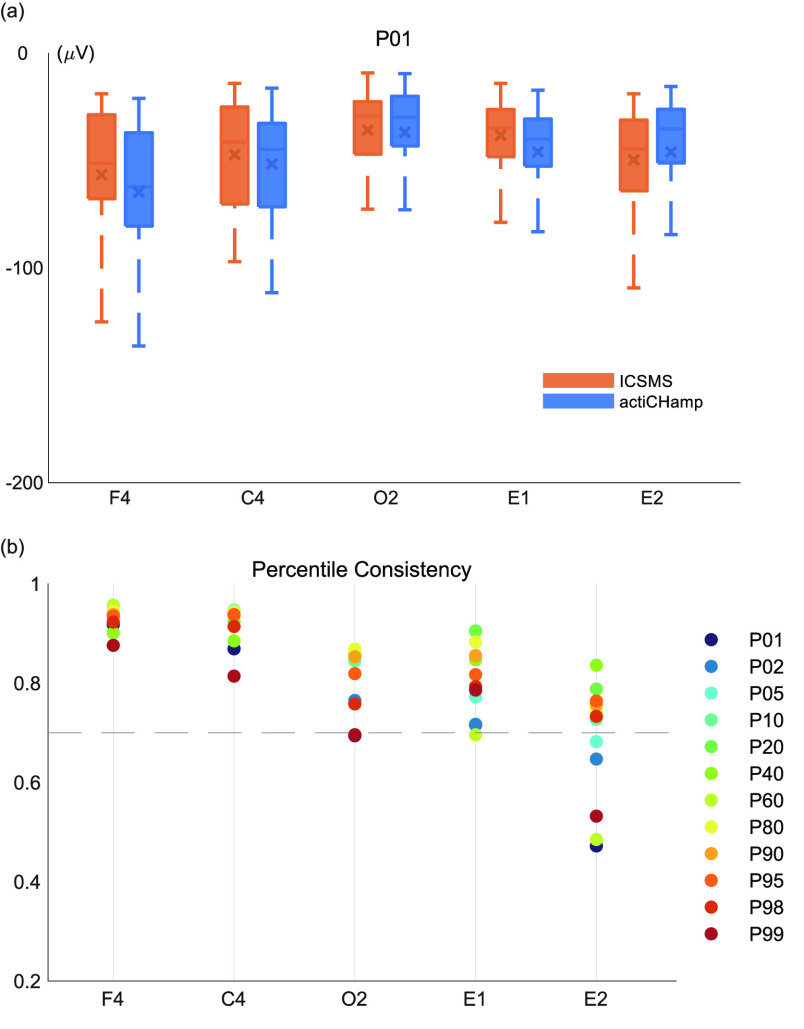
The consistency and distributions of different percentiles. (a) Box plot showing the distribution of the percentiles (P01, shown in Fig. S1. of supplementary materials for the remaining eleven) and (b) scatter diagram showing the ICC of the percentiles for both device data, based on the samples in the above analysis.

Overall, as shown in Fig.6a, all percentiles for the five channels in both devices show the same distribution trend. The ICCs are listed in Fig. 6b. The multiple percentile consistency was greater than 0.75 (with 95% CI = 0.074-0.975; all p < 0.01), with the maximum value reaching 0.957. Moreover, after combining all channel data into a sequence, the consistency value of the percentile distribution reached 0.90 (with 95% CI = 0.754-0.957; all p < 0.0001). However, the consistency among the lower and higher percentiles (P01 and P99) varied only slightly in the E2 channel, most likely due to differences in the EOG electrode position across the two nights. An analysis of the distribution trends of twelve percentiles indicated that the data collected by the two devices were highly consistent.

#### 4) MULTISCALE Entropy in Each Channel

**FIGURE 7.**
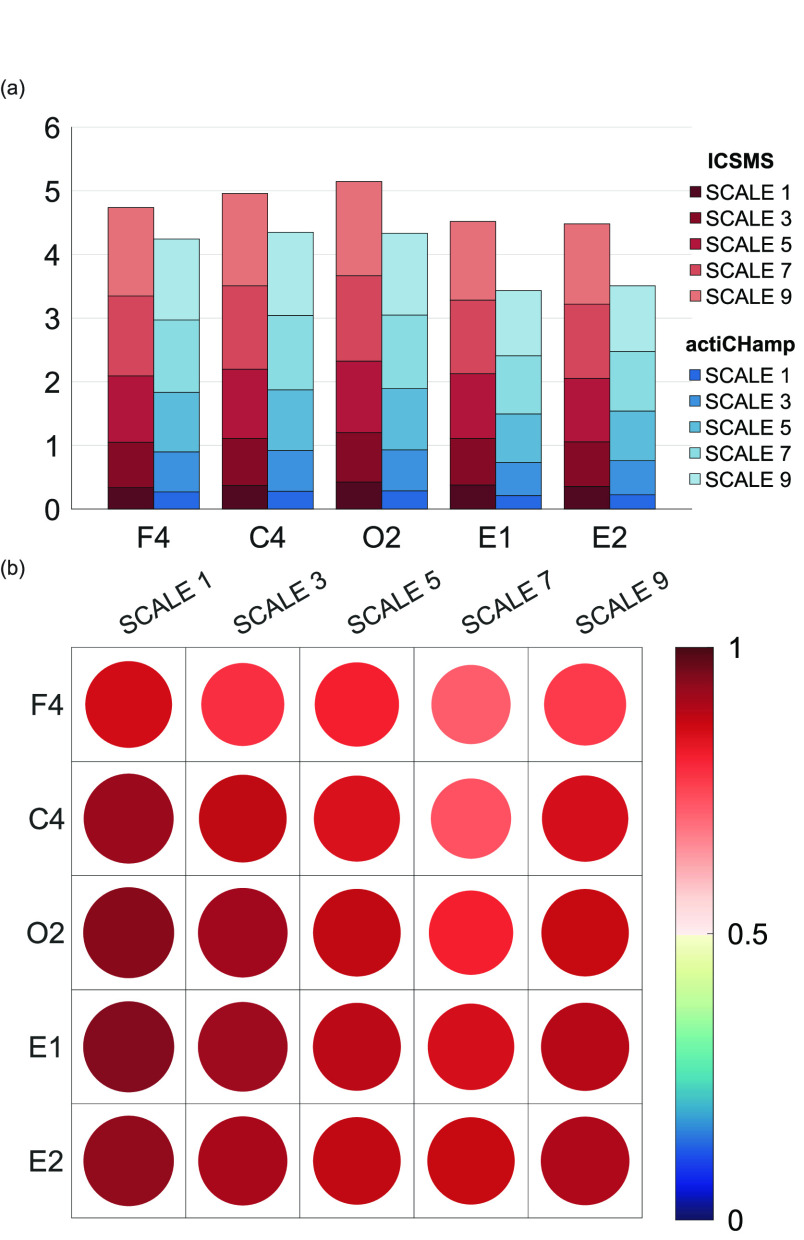
The consistency and trends of the multiscale entropy. (a) Stacked bar chart showing the trends of the multiple reconfiguration dimension feature averages. (b) Within each channel, the consistency among the multiscale entropy at five scales in the two groups data.

The results of the MSE analysis of all channels’ data for both devices are shown in Fig. 7. The entropy values at five scales were averaged and stacked for all participants, and the ICSMS data had slightly larger entropy values than the actiCHamp data, as shown in Fig. 7a. However, the same trends were observed for both groups of data. The ICC values for each device and channel at the five scales are shown in Fig. 7b. The values of scales 1, 3, 5, 7, and 9 were 0.789 ± 0.05 (with 95% CI = 0.505-0.918; all p < 0.001), 0.847 ± 0.07 (with 95% CI = 0.557-0.956; all p < 0.001), 0.885 ± 0.05 (with 95% CI = 0.670-0.968; all p < 0.001),

0.90 ± 0.04 (with 95% CI = 0.745-0.969; all p < 0.001), and 0.898 ± 0.03 (with 95% CI = 0.775-0.962; all p < 0.001), respectively, and most of them were greater than 0.75. Similarly, when all channel data were combined into a sequence, the ICC values of the two groups at the five scales reached 0.812 (with 95% CI = 0.743-0.862; all p < 0.001), 0.882 (with 95% CI = 0.838-0.913; all p < 0.001), 0.913 (with 95% CI = 0.881-0.936; all p < 0.001), 0.915 (with 95% CI = 0.884-0.937; all p < 0.001), and 0.903 (with 95% CI = 0.868-0.929; all p < 0.001). At the single-channel level, the SE showed good inter-device agreement; however, at the single sleep stage level, the results were not satisfactory (0.6334 ± 0.16, with 95% CI = 0.259-0.813; all p < 0.001). This means that the SE of the EEG collected by the two devices had a considerable inter-channel variability induced by the characteristics of the two systems. This also indicates that as a nonlinear dynamic feature, it is difficult to meet the need for higher feature information gain in sleep studies due to poor spatial specificity.

#### 5) So/Spindles in Different Sleep Stages

**FIGURE 8.**
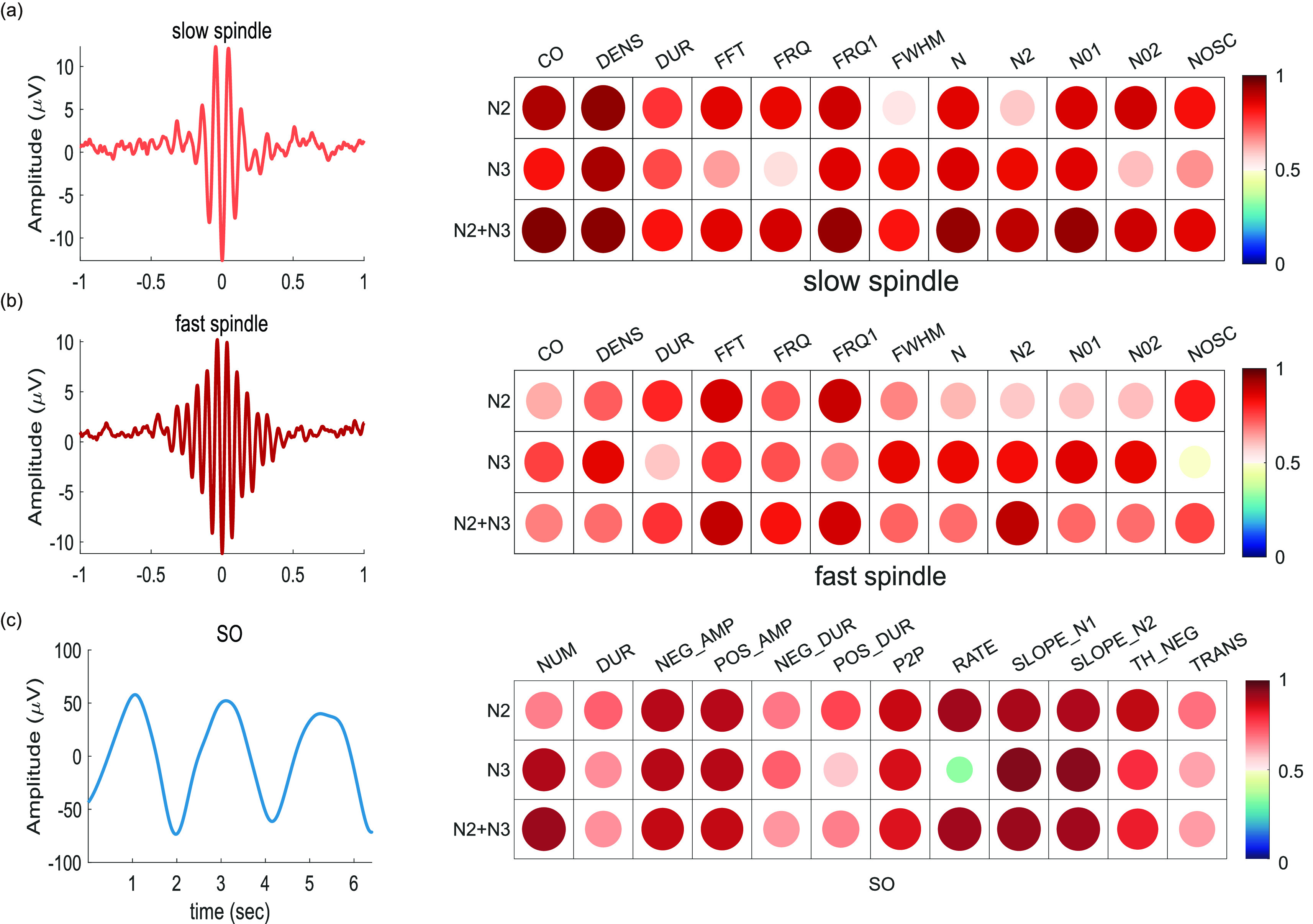
Time domain waveforms (left) and consistency (right) for (a) slow spindles (Fc = 11 Hz), (b) fast spindles (Fc = 15 Hz), and (c) slow oscillations (SOs). The mean EEG signals were time-locked with each spindle peak or slow peak; Morlet wavelets with a central frequency of f0 = 2 were used in the spindle analyses; and the bandpassed (0.5-4 Hz) slow band signal segment are presented to assist with visualization. For the spindle features, CO: couple_overlap, DENS: spindle density, DUR: mean spindle duration, FFT: mean spindle frequency, FRQ: mean spindle frequency, FWHM: full width at half maximum, N/N01/N02/N2: number of spindles, NOSC: mean number of oscillations per spindle. For the SO features, Num: number of SOs, DUR: median SO duration, NEG_AMP: negative peak median amplitude, POS_AMP: positive peak median amplitude, NEG_DUR: negative peak duration, POS_DUR: positive peak duration, P2P: peak-to-peak amplitude, RATE: number of SOs per minute, SLOPE_N1/SLOPE_N2: median slope, TH_NEG: threshold used for negative peak, TRANS: SO transition.

Using the intuitively appealing continuous wavelet transform (CWT) with a Morlet basis function, we identified regions of interest where the power of the CWT coefficients corresponding to the frequencies of slow spindles (9-13 Hz) and fast spindles (13-17 Hz) were large. The detected slow and fast spindles were averaged and synced to the spindle peak (maximum peak-to-peak amplitude). For slow oscillations, the averaged EEG signal was time-locked to the negative peak and included three cycles. Examples of slow and fast spindles are shown in Figs. 8a and b, respectively. In addition, the twelve features extracted from the slow and fast spindle data were statistically consistent between the two devices. As shown in the figure, the ICC values of the slow and fast spindle consistency were 0.827 ± 0.11 (with 95% CI = 0.207-0.976; all p < 0.003) and 0.746 ± 0.10 (with 95% CI = 0.296-0.942; all p < 0.009), respectively. Fig. 8c shows an SO. Similar to the spindle results, the SO features of both devices were statistically consistent, and the SO consistency was 0.793 ± 0.13 (with 95% CI = 0.108-0.969; all p < 0.0001), with a maximum value of 0.948.

### C. Macro Sleep Architecture

The sleep quality of each participant was determined according to the following parameters: the duration of each sleep stage (including N2, N3, and REM); the wake after sleep onset (WASO); the total sleep time (TST); the NREM sleep cycle time (NREMC); the REM latency (REM_LAT) and persistent sleep latency (PER_LAT); and the sleep efficiency (SLP_EFF) and sleep maintenance efficiency (SLP_MA_EFF).

The macro sleep architecture data of the two device groups were compared with Mann-Whitney U tests. Table 2 shows that on the ICSMS and actiCHamp monitoring nights, there were no apparent differences in the macro sleep architecture of the sleep staging determined by the three scorers (p $>$ 0.05 for both; the corresponding U-test is also shown; n = 36). In addition, Table 2 shows the mean and standard deviation of all sleep architecture features for the three scorers’ sleep staging. The average N2, N3 and REM durations in the ICSMS night data were 49.63%, 18.5% and 22.32%, respectively, which is consistent with the range of approximately 50% for N2, 20% for N3, and 25% for REM suggested by the AASM. Moreover, the SLP_EFF values for both nights of sleep scoring were greater than 92%, and the SLP_MA_EFF was greater than 95%, indicating that all participants (except for two, who were not scored) had normal sleep nights and that the ICSMS device was sufficient for in-home sleep monitoring, as expected.

**TABLE 2:** Significance and mean ± SD for macro sleep architecture metrics.

	Mann-Whitney(p value and U)				Statistics value					
	(ICSMS-actiCHamp)				Scorer 1		Scorer 2		Scorer 3	
	Scorer 1	Scorer 2	Scorer 3	Device	Mean	SD	Mean	SD	Mean	SD
N2	0.279	0.839	0.563	ICSMS	48.83%	6.75%	48.87%	9.16%	60.77%	41.03%
proportioon	(U=197)	(U=169)	(U=143)	actiCHamp	45.52%	7.05%	48.69%	6.68%	52.51%	5.33%
N3	0.085	0.091	0.051	ICSMS	18.77%	6.88%	19.30%	10.33%	15.07%	6.74%
proportioon	(U=107)	(U=108)	(U=224)	actiCHamp	24.88%	9.73%	23.71%	7.55%	10.25%	6.07%
REM	0.059	0.864	0.584	ICSMS	22.45%	4.16%	23.47%	7.78%	21.08%	7.18%
proportioon	(U=222)	(U=168)	(U=180)	actiCHamp	19.80%	4.69%	23.65%	4.94%	20.83%	4.91%
NREMC	0.542	0.501	0.443	ICSMS	100.97	24.23	105.23	27.90	99.11	23.77
(mins)	(U=142)	(U=183)	(U=187)	actiCHamp	100.07	15.18	97.04	19.33	91.66	14.68
WASO	0.097	0.888	0.696	ICSMS	17.25	11.15	11.75	11.14	22.81	17.23
	(U=109.5)	(U=157)	(U=175)	actiCHamp	23.17	14.05	13.11	17.28	19.64	15.19
TST	0.719	0.743	0.963	ICSMS	467.64	63.24	471.91	66.63	462.92	63.81
(mins)	(U=173)	(U=172)	(U=163.5)	actiCHamp	459.63	59.49	468.36	59.17	462.50	60.44
PSL_LAT	0.323	0.484	0.563	ICSMS	12.19	8.62	10.55	7.04	12.86	8.61
(mins)	(U=130.5)	(U=139)	(U=143.5)	actiCHamp	16.89	13.22	15.22	12.98	16.47	13.27
REM_LAT	0.743	0.628	0.443	ICSMS	71.25	47.03	80.00	55.85	84.11	52.14
(mins)	(U=151)	(U=178)	(U=187)	actiCHamp	73.83	40.88	68.11	39.04	68.22	39.84
SLP_EFF	0.091	0.252	0.650	ICSMS	93.34%	4.61%	95.30%	3.49%	92.67%	5.08%
	(U=216)	(U=199)	(U=177)	actiCHamp	88.63%	10.66%	94.18%	4.49%	92.59%	4.4%
SLP_MA_EFF	0.085	0.888	0.839	ICSMS	96.34%	2.57%	97.39%	2.82%	95.12%	4.11%
	(U=217)	(U=166.5)	(U=155)	actiCHamp	95.16%	2.87%	97.28%	3.56%	95.88%	3.13%

p>0.05 represent no significant difference, the Mann-Whitney U-test are shown in’ (U=xxx)’.

## V. Discussion

This study demonstrates a novel in-home sleep monitoring system and its multilevel analysis. The multilevel analysis includes multi-scorer sleep staging results, micro sleep architecture characteristics, and macro sleep architecture characteristics. Firstly, cross-validation analysis of the sleep staging results using three specialized physicians for both data groups can avoid subjective staging errors due to individual reasons, thus improving the credibility of the results. Secondly, the micro sleep architecture addresses the time domain (percentiles, SO/Spindle), frequency domain (power-law, RELPSD), and nonlinear features (MSE). Among them, PSD provides a more objective alternative perspective for the analysis of EEG signals and reveals the activity state of the brain during sleep activity [39]. In particular, the RELPSD reveals the physiological significance of different frequency bands of EEG during different sleep stages in a relative form. In terms of a neurophysiological mechanism, the PSD slope varies with the strength of temporally correlated population spiking activity, being steeper when neural activity is more synchronized during sleep [40]. It has also been shown that MSE, a complex analysis of biological signals, can identify dynamic changes in surface EEG [41], and reflect the balance between sleep and alertness facilitators [38]. In addition, percentiles are descriptive analyses of raw data, which provide better estimates when based on grouped data than raw data [42]. Spindles predominate in the central and parietal brain regions, which studies have shown to be correlated with sleep-associated performance improvements on declarative tasks [43]. A previous study also proposed that cortical SO drives spindle activity [44] and that the coordination of SO and spindle activity during NREM sleep plays a crucial role in sleep-dependent memory consolidation and other sleep regulations [45]. Finally, macro sleep architecture is generally considered as sleep cycles and stage sequences throughout the night, monitoring sleep stages and analyzing sleep structure are among the main approaches for diagnosing sleep disorders and assessing the effect of treatment [46], [47]. We believe that the analysis and validation results, including both subjective and objective consistency metrics, provide reliable evidence indicating the clinical translation and research utility of this generation of devices.

However, the proposed system also has certain limitations. Firstly, the need for a professional technician to operate the system, which cannot be done independently by the participant’s family with simple training. Secondly, the data acquisition and analysis were performed on 20 healthy participants, but the inclusion of broader participants (not only young healthy participants), especially on a more balanced gender and patients with sleep respiratory disorders, is a necessity in future work in order to test the performance of this system on patients with sleep disorders in the real environment. Thirdly, more and more direct feature analysis needs to be compared with PSG for a more comprehensive assessment of the system. Finally, in the follow-up work, we will continue to optimize the chip circuit performance, reduce the system power consumption to improve endurance, optimize the structure design, simplify the operation steps to improve the portability of the system and provide a more comfortable user experience.

Currently, there are no standard portable in-home sleep monitoring systems with multichannel EEG signal acquisition or enforceable system performance parameter guidelines. We believe that an in-home sleep monitoring system needs to provide not only excellent performance at the circuit and system level, but also overall stability improvement after multilevel evaluation tests. The demonstration of a novel in-home sleep monitoring system in this paper emphasizes a comprehensive consistency or significance test of in-home sleep data with an advanced system, including a comparison of the micro sleep architecture of each channel, at each stage throughout the night. We believe that “in-home” monitoring is a broad need for people, especially in the daily physiological process of sleep. This means that, even with the rapid development of electronic technologies, there is still a need for developers to keep in mind the need to test technologies in the home environment, where real physiological processes are prevalent, to facilitate the diffusion and optimization of electronic devices. We suggest that going forward, in a multichannel EEG-based portable in-home sleep monitoring system, performance parameters need to be achieved as followed: IRN $< 1\mu $Vpp, SNR $>$ 100dB, CMRR $>$ 110, DR $>$ 110dB, Input impedance $>$ 1G $\Omega $, weight $< 60\text{g}$, and with an endurance life greater than 24 hours. Meanwhile, in the future, researchers developing in-home sleep monitoring systems or technologies should consider the analytical framework of this paper as a starting point. While improving the performance of the physiological signal acquisition circuit, gives a stable and reliable evaluation report of real in-home sleep monitoring in multilevel sleep data analysis.

## VI. Conclusion

This paper is the first publicly reported portable sleep monitoring system based on a fully integrated multichannel front-end chip for in-home use. Based on the strict requirements of in-home systems, we developed an eight-channel biopotential acquisition front-end chip with low noise, high CMRR, high DR, and high input impedance, as well as trade-off size, weight, endurance, and signal quality requirements, and incorporated this chip into the system proposed in this paper. Compared with the data collected by a standard PSG system, this system showed high consistency or no significant differences in multi-scorer sleep staging results (e.g., kappa coefficients), micro sleep architecture (e.g., RELPSD, slowing index, MSE, percentiles, SO/spindle characteristics), and macro sleep architecture (e.g., N2/N3/REM duration, TST, SLP_EFF). Certain statistical results also indicate that the experimental data collected by this system outperform data acquired by commercial PSG, suggesting that the system can be used for long-term in-home sleep monitoring. In future work, we will continue to use this system in a variety of populations (including healthy participants of different ages and genders and patients with sleep disorders) and the real environment to provide more evidence and support for the clinical translation and research utility of this generation of devices.
